# Development of a New Grading Scale for Evaluating Overall Hair Density

**DOI:** 10.1111/jocd.70710

**Published:** 2026-02-06

**Authors:** Wanhua He, Junwei Tang, Jiahui Pan, Yanrui Gao, Wencai Jiang, Yimei Tan

**Affiliations:** ^1^ Department of Skin and Cosmetic Research Shanghai Skin Disease Hospital, School of Medicine, Tongji University Shanghai P.R. China; ^2^ Professional Technical Service Platform for Clinical Evaluation of Skin Health Related Products Shanghai Science and Technology Commission (21DZ2294500) Shanghai P.R. China; ^3^ NMPA Key Laboratory for Monitoring and Evaluation of Cosmetics Shanghai P.R. China

## Abstract

**Background:**

Hair density influences overall attractiveness significantly. Although pre‐existing classifications have been used to describe alopecia pattern and evaluate treatment efficacy, their accuracy is undesirable.

**Objective:**

The aim of this study is to develop a stepwise and accurate graded visual scale regardless of sex and verify its application value in response to treatment efficacy.

**Methods:**

Based on 6644 standard images, the seven‐point grading scale for overall hair density was developed to describe alopecia extent. Reliability and validity studies were conducted to identify intra‐rater and inter‐rater consistency. Image J and Dermoscopy were introduced to quantify macro‐ and micro‐hair characteristics and determine the gradient difference of the classification system. The sensitivity to treatment response was verified by one model trial to evaluate its application value.

**Results:**

The intra‐rater and inter‐rater reliability revealed excellent agreement and consistency of the severity grading, independent of the time of evaluation and evaluator. Moreover, quantitative hair characteristics, including global exposed scalp area ratio, hair diameter, local hair density, vellus hairs ratio and vellus to terminal hairs ratio, were closely related to the corresponding grades. In the model trial, overall hair density evaluated by this scale has significantly improved in the test group, while there was no obvious change in the control group. The observed trend of the evaluation result was consistent with changes in the global exposed scalp area ratio and local hair density.

**Conclusions:**

The new grading scale represents a reliable and objective indicator for a valid, reproducible, and sensitive evaluation of alopecia extent and treatment response.

## 1 Introduction

Alopecia is a common dermatologic condition, which affects up to 50% among people over the course of a lifetime [1]. Although hair loss is often considered a benign medical condition, it has a dramatic effect on psychological stress [2]. Current treatment options for alopecias mainly include oral finasteride, topical minoxidil, and hair transplantation; other available therapies with minimal adverse events are still explored [3]. Importantly, how to evaluate the efficacy is of great significance for the development of new treatment strategies. Based on the above high prevalence and strong demand in efficacy evaluation, one alopecia classification scale that is sensitive to extent and treatment response must be developed.

The development of a classification scale depends on the hair loss pattern determined by differences in androgen‐sensitivity of the scalp areas. Currently, the most widely used grading scale for male androgenetic alopecia is the Hamilton–Norwood classification, established in 1975 and subdivided into seven stages and special types IIa–Va [4]. In 1977, Ludwig suggested one 3‐scale classification for female‐pattern hair loss (FPHL) [5]. Later, the works done by Savin and Sinclair introduced more detailed scales to highlight FPHL [6]. In daily clinical practice, the Savin scale requires comparative matching of the patient's hair parted along the mid‐line to the 8 computer‐generated pictorial representations of the central scalp hair parted in the middle [7]. The 5‐point Sinclair scale was further modified to evaluate the FPHL degree in men [8]. Until 2007, the basic and specific classification (BASP) was developed as a gender independent scale, which was based on common features of the balding process including the recession of the anterior hairline and reduction of hair density on the crown and vertex region [9]. Jin et al. in 2018 proposed a semi‐quantitative classification system, which showed the gradient difference at each grade through objective data by dermoscopy and image analysis [10].

Although pre‐existing classifications have been used to describe hair loss patterns and evaluate treatment efficacy, their accuracy is undesirable due to the wide gap between each grade applied for evaluation in clinical diagnosis [7]. When using the existing classification methods, hair density achieves improvement from one grade to the next, which typically occurs in the course of a 6‐ to 12‐month clinical trial of a hair‐growth promoter, leading to a long trial period that is unfavorable for describing the real trend of hair loss [7]. Early recognition and treatment are important, which can help to halt its progression and preserve hair as much as possible [11]. However, we noticed limitations of published classifications on the diagnosis of early‐stage hair loss. Existing scales often have broad grading intervals, which limit their applicability to distinguish minor differences among the hair density characteristics of early cases. Therefore, the development of a new sensitive classification scale, especially for mild‐to‐moderate alopecia extent and early treatment response, will be of great significance.

The present work aims to establish a new classification system to reduce the gap in alopecia extent, which is sensitive and stepwise regardless of gender. Additionally, quantitative methods were introduced to explore the most frequent characteristics of hair loss and verify the gradient between each grade. Considering hairline change is unremarkable, we focus exclusively on the grade of overall hair density on the frontal and vertex region, characterized by a high expression of androgen receptors.

## 2 Methods

### 2.1 Participants

This is a retrospective study that involves a total of 1661 subjects who participated in clinical trials for evaluating the efficacy of anti‐hair loss dermatological products between January 2020 and January 2025. The main inclusion criteria were self‐reported excessive hair loss and/or slight hair thinning. Other inclusion and exclusion criteria are shown in Table S1, all of which complied with the efficacy measurement method of anti‐hair loss cosmetic products in China Safety and Technical Standards for Cosmetics. The investigation is approved by the institutional ethics committee.

### 2.2 Image Acquisition

Grading scale creation was based on a huge photo database obtained over more than 4 years. Photographic images of the vertex (V) + frontal (F) aspect of the scalp were collected with a 24 megapixels camera (Nikon D610) at a single center. All standardized photographs were obtained in the same room with the parameters including aperture, ISO, shutter speed, focal distance, white balance, and distance between the camera and the subject kept invariable. To ensure the quality and consistency of the images, all subjects were informed not to wash their hair within 48 ± 4 h before the visit. After 30 min of acclimation under controlled conditions with environmental temperature of 20°C–22°C and related humidity of 40%–60%, the hair of all subjects was combed along the mid‐line to the sides before taking pictures. 6644 photographs were collected across 4 visits per subject to grade overall hair density throughout the clinical trial.

### 2.3 Scale Development and Reliability Examination

Standard images were cropped horizontally from the bottom edge of the standard color card to the upper edge of the head and vertically closely to the head on the left and right sides to produce images of the area of interest. Photograph assessment was independently performed by three trained raters. 6644 photographs without personal information were provided. They divided these photographs into seven grades. Representative images were selected for each grade to complete standard grade images.

100 photographs with different hair density were evaluated by three raters independently according to this grade scale, which was repeated 4 weeks later. The order of the photographs had been randomly disturbed before every assessment to eliminate recall bias.

### 2.4 Gradient Difference Validation

According to this new scale, 120 hair photographs were selected randomly from the image database and graded by two raters. Corresponding global area ratio of exposed scalp and local hair information was also analyzed to identify relevance between grade result and those quantitative data.

The grayscale values of scalp and hair on one photo have significant differences. Therefore, digital photos of V + F area were imported into Image J software to quantify overall alopecia extent, while the photo size and analysis area should keep fixed during analytical process. Area ratio of exposed scalp was calculated. The exposed scalp area ratio was defined as the proportion of visible scalp area relative to the total analyzed area in the V + F region, with higher values indicating lower hair density and worse alopecia.

Molemax dermoscopy is a useful tool to obtain high‐definition photographs of local hair. For all subjects, 1.5 cm × 1.5 cm of targeted area was marked with transparent film (the temporal side adjacent to the vertex of the head) and shaved, and the length of residual hair was less than 1 mm. Local hair density (n/cm^2^), hair diameter (μm). Additionally, the ratios of vellus hairs (%) and terminal hairs (%), as well as the vellus‐to‐terminal hair ratio were calculated by Trichoscan software. A vellus hair is thinner than 40 μm, while a terminal hair is thicker than 40 μm. The ratio of vellus hairs and terminal hairs referred respectively to the percentage of each hair type relative to the total hair number within the analyzed area. The vellus‐to‐terminal hair ratio was defined as the numerical ratio of vellus hairs to terminal hairs.

### 2.5 Sensitivity of Grade Scale to Treatment Response

A previously conducted clinical trial in our center demonstrated the efficacy of anti‐hair loss product, as evidenced by local hair density and hair loss counting. This study, which started in February 2024 and ended in June 2024, was developed in accordance with method for efficacy measurement of anti‐hair loss cosmetic products in China Safety and Technical Standards for Cosmetics. All subjects used the control products 3 times per week during a 2‐week washout period firstly; during the formal study, test and control group were assigned to apply either the investigation product (IP) or the control product (CP) 3 times per week for 12 weeks. The flow chart was shown in Figure S1. Clinical assessment and noninvasive instrument measurement are performed before the study products application (Baseline, BL) and at Week 4, Week 8, and Week 12 after the study products application. The demographic information and preliminary instruments results in this trial were detailed in Table S2 and Figure S2.

Therefore, we chose this study as a model to explore the changing trends of both the test and control groups under this clinical grade scale. All hair digital images at every visit were retrospectively evaluated by two raters to verify scale sensitivity to treatment response, while raters were blinded to treatment types and timepoints. The exposed scalp area ratio was also calculated.

### 2.6 Statistical Analysis

Data were analyzed using the SPSS statistical software 22.0 for Windows. Descriptive analysis was performed for each parameter including mean, standard deviation or median, minimum, and maximum depending on data type. Kolmogorov–Smirnov test was performed for normality test of quantitative data to determine whether data were parametric or non‐parametric analysis. The Kappa was calculated to determine the intra‐rater reliability (for the same evaluator at 2 different time points) and Fleis‐Kappa was used for the inter‐rater reliability (consistency of the 3 raters) to determine. Spearman correlation coefficient was used to calculate whether data were statistically correlated with grade and instrument measurements. According to data distribution, datasets were analyzed using one of the two following methods for comparison of between before and after application: Paired Student *t*‐test and Wilcoxon Signed Rank test. For comparison of the net change between the test and the control product, Independent Samples *t*‐test and Independent Samples Wilcoxon Signed Rank test were used. All statistical tests were 2‐sided at significant level with *p* < 0.05.

## 3 Results

### 3.1 Participants

This study involved 1661 subjects (440 males and 1221 females) with 6644 images. The demographic distribution of the subjects is listed in Table 1. The average age in both sexes was 47.57 ± 9.77 years (range 18–60). Most of the subjects were aged 51–60 years, followed by 41–50 years.

**TABLE 1:** Demographic distribution.

Age group	Age range	Average age	Total
1	18–30	26.52 ± 3.06	108
2	31–40	36.36 ± 2.74	294
3	41–50	45.63 ± 2.87	502
4	51–60	55.95 ± 2.90	757
Total	18–60	47.57 ± 9.77	1661

### 3.2 Scale

**FIGURE 1:**
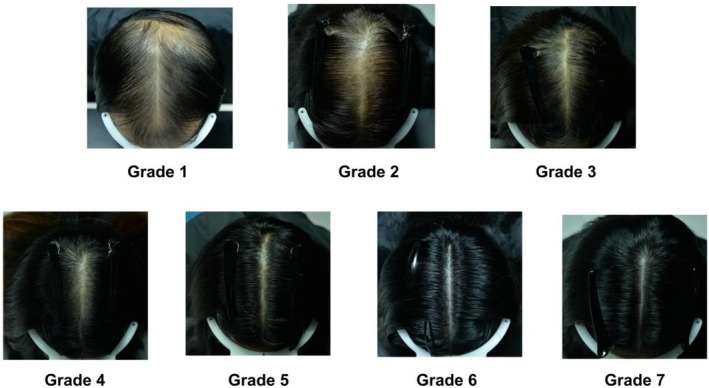
The grading scale of the vertex and frontal area.

The standard scale of alopecia ranged from 1 to 7, which sets graded photographs involving the V + F area, as listed in Figure 1. In brief, from grade 1 to 7, an increase in the degree of hair density can be observed.

As shown in Table 2, the intra‐rater reliability analysis at the 2 assessments performed by each observer revealed an excellent correlation and consistency of the grading severity, independently of the evaluation time, with Kappa coefficient range of 0.971 to 0.990 (*p* < 0.001). The inter‐rater reliability analysis revealed Fleiss‐Kappa coefficient of 0.838 (*p* < 0.001) in Table 2.

**TABLE 2:** Descriptive statistics of the intra‐rater and inter‐rater reliability.

Intra‐rater reliability			
Rater	Kappa statistic	*p*	Kendall's coefficient
1	0.980	< 0.000	0.986
2	0.990	< 0.000	0.994
3	0.971	< 0.000	0.977

Inter‐rater reliability			
Rater	Fleiss‐Kappa statistic	*p*	Kendall's coefficient
1 versus 2 versus 3	0.838	< 0.000	0.960

### 3.3 Correlation Between Grading and Quantitative Hair Parameters

**FIGURE 2:**
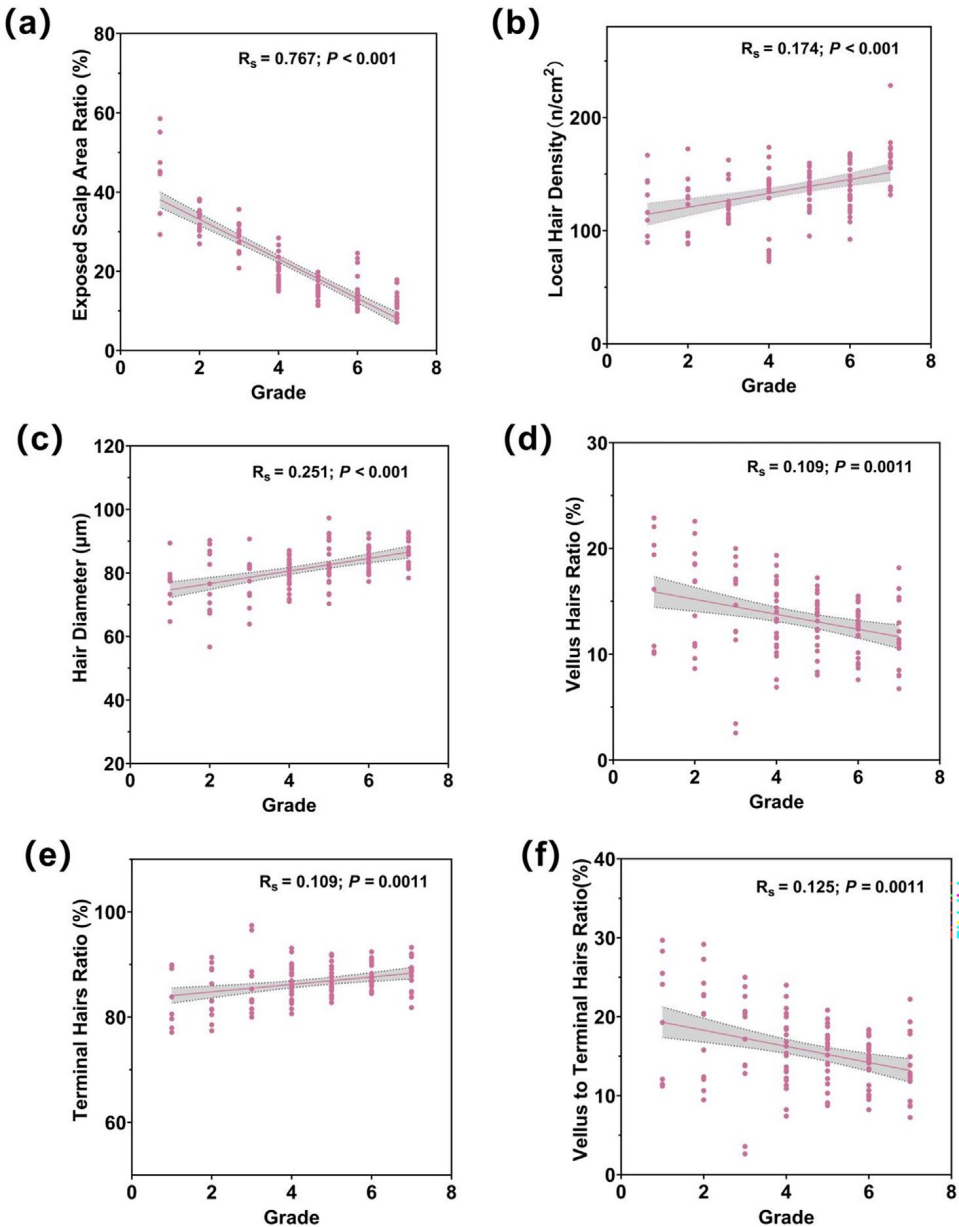
Correlation between variables. (a) Grade scores and Exposed scalp area ratio. (b) Grade scores and Hair diameter. (c) Grade scores and Local hair density. (d) Grade scores and Vellus hairs ratio. (e) Grade scores and Terminal hairs ratio. (f) Grade scores and Vellus to Terminal hairs ratio. (*R*_*s*_ = Spearman's rank correlation coefficient; with corresponding *p* values).

Based on the gradation standards, overall hair density degrees of 120 subjects were determined. The results of quantitative hair parameters in different grade were shown in Table S3. Our results indicated that the exposed scalp area ratio, vellus hairs ratio and vellus to terminal hairs ratio was negatively correlated with grading (*p* < 0.01, Figure 2). These grading scores were also found to be increased with increasing local hair density, hair diameter, and terminal hairs ratio (*p* < 0.01, Figure 2).

### 3.4 Grading Scale Application in One Clinical Trial

To determine grading scale sensitivity to treatment response, all hair images from the clinical trial were retrospectively graded. The evaluation results were listed in Tables 3.1 and 3.2. Compared with BL, the grading of overall hair density increased significantly at Week 12 after the investigation product application (*p* < 0.001), which showed no significant difference at any time point after the control product application (*p* > 0.05). There were significant differences in the net change of overall hair density from BL between the investigation product and the control product at Week 12 (*p <* 0.01). Representative photographs before and after test product application were shown in Figure S3.

**TABLE 3.1:** The overall hair density evaluation from different groups at different time points [median (min, max)].

Group	Visit	
	IP	CP
Baseline	5.8 (3, 7)	6 (4, 7)
Week 4	6 (3, 7)	6 (4, 7)
Week 8	5.5 (3, 7)	6 (4, 7)
Week 12	6 (3.5, 7)***	6 (4, 7)

Abbreviations: CP, control product; IP, investigation product.

*** Significantly different from baseline, *p* < 0.001.

**TABLE 3.2:** The net change of overall hair density from different groups at different time points [median (min, max)].

Group	Visit	
	IP	CP
△ Week 4	0 (−0.5, 1)	0 (0, 0.5)
△ Week 8	0 (0, 1)	0 (−1, 0.5)
△ Week 12	0 (0, 0.5)**	0 (0, 0.5)

Abbreviations: CP, control product; IP, investigation product.

** Significantly different from CP, *p* < 0.01.

**FIGURE 3:**
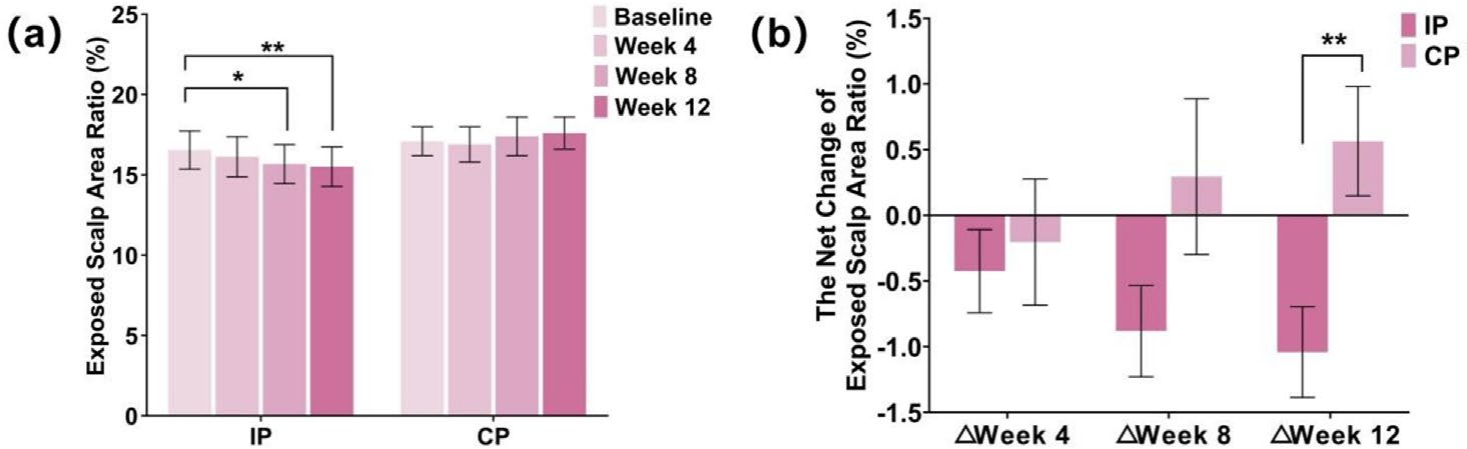
The results of the exposed scalp area ratio in the model trial. (a) The values from different groups at different time points. (b) The net change values from different groups at different time points. Data are presented as Mean ± SD. CP, control product; IP, investigation product. *p < 0.05, **p < 0.01.

The exposed scalp ratio analyzed by hair images cooperatively verified scale sensitivity; the results were listed in Tables S4.1 and S4.2 and Figure 3. Compared with BL, the exposed scalp area ratio decreased significantly at Week 8 and Week 12 after the investigation product application (*p* < 0.05), which showed no significant difference at all time points after the control product application (*p* > 0.05). There were significant differences in the net change of exposed scalp ratio from BL between the investigation product and the control product at Week 12 (*p <* 0.01).

## 4 Discussion

In the present study, we have developed and validated a scale to standardize overall hair density that may be useful in daily clinical practice to establish scientific public awareness, standardize clinical evaluations, guide treatment, and assess treatment outcomes systematically for patients care and research purposes as well. The advantage of this scale lies in ensuring high consistency and repeatability while achieving high sensitivity and breaking the gender differentiation of traditional scales. In both sexes, hair loss typically involves the frontal and vertex regions, characterized by high expression of androgen receptors [12]. Therefore, establishing a unified classification method for both sexes focusing on hair density change of the above regions is both reasonable and practical during daily clinical practice or scientific research.

High Fleiss–Kappa values and Kendall's coefficients confirmed an excellent agreement among the raters, and an internal consistency independent of the rater. The scale showed good consistency and reproducibility for esthetic evaluation of overall hair density, ranging from Grade 1 (extremely sparse hair) to Grade 7 (extremely dense hair).

To determine the gradient difference in this grading scale, 120 photographs were graded by two raters using the grading scale, while local and overall hair parameters were quantified by Dermoscopy and Image J. It has been reported that the prevalence information of Dermoscopy parameters represents an accurate clinical sign from the local area, improving the diagnostic capability [13]. The decrease in hair density and terminal hair ratio is related to the increase of alopecia severity in both sexes [12]. Moreover, one threshold segmentation method was applied, using grayscale images to effectively distinguish scalp from hair for quantification of overall hair density. In this study, evaluation results were compared and analyzed to identify the high relevance of grade to biophysical parameters, including exposed scalp area ratio, hair density, vellus hairs ratio, terminal hairs ratio and vellus to terminal hairs ratio. The gradient difference in this grading scale was further illustrated according to the analyzed images by Image J (Figure S4). Therefore, the new grading scale was exploitable as one of objective criteria to describe the alopecia degree.

Given the proven validity, reliability, and gradient of the hair density scale, this study applied this scale for both the grading of alopecia severity before treatment and the assessment of treatment outcomes, which effectively evidenced its high sensitivity. Previously developed scales are BASP, Hamilton–Norwood, and Ludwig, etc., but the common defect is the wide gap between different grades. The treatment innovations, including new drug development, hair transplantation, and laser therapy, underlined the need to have one sensitive scale in positive response to efficacy of these strategies. A complete clinical trial was selected as the model during the scale validation phase. This study indicates that alopecia severity evaluated by this scale in the test group has significantly improved while there was no obvious change in the control group. The changing trend of evaluation results is consistent with those of local dermoscopy parameters and global exposed scalp area ratio. Additionally, we have further evaluated all images in the model trial by Sinclair Scale for female and Modified Sinclair Scale for male. Both the test group and control group showed no significant evaluation difference at all time points after product application (*p* > 0.05, Table S5), in which data of 15 participants were excluded in the statistics analysis as their alopecia levels were graded below 1 on these scales. The above results clearly indicated that existing classification systems have limitations to distinguish minor differences among the hair density characteristics of early‐stage hair loss. Additionally, these results also demonstrated the low sensitivity of these classification systems to treatment response. Although Jin et al. conducted similar validation to describe their grading scale sensitivity, only one case was investigated, which was insufficient to support effective evidence [10]. Unlike traditional scale development, this is the first study to systematically verify sensitivity of one new alopecia scale to treatment response.

The high sensitivity greatly enhances the application value of the grading scale, especially in clinical treatment and trials. The major problem in the available grading scale is that the change in hair density that leads to a clear increase from one pattern to the next typically occurs in the application of 6‐ to 12‐month hair‐growth promoters for clinical trial [7]. Hair density evaluation by grading scales is probably not suitable as an end point in clinical trials. Our rating scale seems to be a more practical solution for use aiming to accurately capture change in the early treatment stage and shorten the clinical trial cycle. In recent years, cosmetic treatment has been proved as a good auxiliary modality to regulate the signaling pathway of hair growth, improve the scalp micro‐environment, activate dermal papilla stem cells, and stimulate hair growth and regeneration [14, 15]. Their effects are modest and cannot serve as replacements for pharmacological therapy; therefore, current scales are less suitable for assessing hair density of cosmetic treatment. High sensitivity proved in one clinical study also indicates an extensive prospect of our scale to measure the efficacy of cosmetic treatments. It is worth noting that in 2021, China Safety and Technical Standards for Cosmetics proposed efficacy measurement of anti‐hair loss cosmetic products that requires manufacturers to demonstrate product efficacy in order to protect subjects from misleading claims. Until now, this method only provides text descriptions for the overall hair density grading standard ranged from 1 to 7, lacking a legend that reduces the consistency and accuracy during the evaluation process (Table S6). Our work can compensate for the insufficiencies of the above methods and provide a reference tool for the cosmetic industry.

Great strides have been made during the last decades in the evaluation methodology of hair loss, while dermoscopy is considered as revolutionized the approach to quantify hair information involved hair density, hair growth rate, vellus and terminal hairs ratio, etc. [16] Its limitation is too small a field of vision, which only reflects local changes. Both photographic and live rating might be preferable options to determine overall hair condition, and the development of a standard scale is the base of evaluation objectivity. Therefore, our grading scale is an important tool to accurately reflect alopecia extent and treatment response regardless of sex, and has broad application prospect to assess public hair health.

Although this study developed one new sensitive grading scale to describe alopecia extent, there are still limitations to address. First, as an effective supplementary approach to traditional grading scales, it is necessary to clarify that our work aims to subdivide alopecia symptoms for mild‐to‐moderate patients, not for late‐stage patients. Additionally, the samples in this study were restricted to the Chinese region, and individuals with curly hair were also excluded, which limits generalizability to diverse regions, countries, ethnicities, and curl patterns. In future studies, we will try to conduct multicentric and trans‐regional clinical trials to validate the applicability of our grading scale for populations with varied races and hair curl patterns.

## 5 Conclusion

Based on real images and hair parameters, this work represents a valid, objective, and accurate 7‐point scale for the evaluation of overall hair density, which can be specifically applied to Chinese men and women with straight hair. The validation experiments showed excellent validity and reliability of this grading scale. This scale, with high sensitivity to alopecia severity and treatment response, seems to be a valuable tool to provide a validated reference in daily clinical practice and aesthetic research.

## Author Contributions

**Wanhua He:** writing – original draft, methodology, validation, investigation. **Junwei Tang:** investigation, validation, data curation. **Jiahui Pan:** investigation, data curation. **Yanrui Gao:** validation. **Wencai Jiang:** conceptualization, writing – review and editing, supervision. **Yimei Tan:** conceptualization, writing – review and editing, supervision, project administration.

## Ethics Statement

The Ethics Committee of Shanghai Skin disease Hospital approved this study (approved No. 2021‐152).

## Consent

Informed consent was obtained from all patients involved in this study.

## Conflicts of Interest

The authors declare no conflicts of interest.

## Supporting information


**Table S1:** Inclusion and exclusion criteria.
**Table S2:** Demographic data of the model trial.
**Table S3:** The results of quantitative hair parameters in different grades (Mean ± SD).
**Table S4.1:** The exposed scalp ratio from different groups at different time points (Mean ± SD, %).
**Table S4.2:** The net change of exposed scalp ratio from different groups at different time points (Mean ± SD, %).
**Table S5:** The overall hair density evaluation graded by the Sinclair Scale for female and the Modified Sinclair Scale for male from different groups at different time points.
**Table S6:** Overall hair density grading standard in the “China Safety and Technical Standards for Cosmetics”.
**Figure S1:** Flow chart in the model trial.
**Figure S2:** Instruments results in the model trial. (a) The hair loss counting and (b) The local hair density from different groups at different time points. (c) The net change of hair loss counting and (d) The local hair density from different groups at different time points. Data are presented as Mean ± SD. CP, control product; IP, investigation product. *p < 0.05, **p < 0.01, ***p < 0.001.
**Figure S3:** Representative photographs (subject No. A18) before and after test product application. (a) Digital live photo. (b) Analyzed photo by Image J (up at Baseline and down at Week 12).
**Figure S4:** The grading scale developed in this study analyzed by Image J.

## Data Availability

The data that support the findings of this study are available from the corresponding author upon reasonable request.
