# Supplementary material for: Development of a New Grading Scale for Evaluating Overall Hair Density
Source: J Cosmet Dermatol. 2026 Feb 6;25(2):e70710. doi: 10.1111/jocd.70710 (PMC12881707; doi:10.1111/jocd.70710)
Supplement: Supplementary file 1 — Table S1: Inclusion and exclusion criteria. Table S2: Demographic data of the model trial. Table S3: The results of quantitative hair parameters in different grades (Mean ± SD). Table S4.1: The exposed scalp ratio from different groups at different time points (Mean ± SD, %). Table S4.2: The net change of exposed scalp ratio from different groups at different time points (Mean ± SD, %). Table S5: The overall hair density evaluation graded by the Sinclair Scale for female and the Modified Sinclair Scale for male from different groups at different time points. Table S6: Overall hair density grading standard in the “China Safety and Technical Standards for Cosmetics”. Figure S1: Flow chart in the model trial. Figure S2: Instruments results in the model trial. (a) The hair loss counting and (b) The local hair density from different groups at different time points. (c) The net change of hair loss counting and (d) The local hair density from different groups at different time points. Data are presented as Mean ± SD. CP, control product; IP, investigation product. *p < 0.05, **p < 0.01, ***p < 0.001. Figure S3: Representative photographs (subject No. A18) before and after test product application. (a) Digital live photo. (b) Analyzed photo by Image J (up at Baseline and down at Week 12). Figure S4: The grading scale developed in this study analyzed by Image J. [file JOCD-25-e70710-s001.doc]

**Table S1.** Inclusion and exclusion criteria.

| Inclusion criteria |
| --- |
| 1. Healthy males or females aged 18~60 years; |
| 1. Subjects’ hair length was between 5 cm~40 cm; |
| 1. Subjects were self-reported to excessive hair loss and/or slight hair thinning, and the number of hair loss > 10 by means of the method of 60 times combing at screening visit and baseline visit; |
| 1. Subject had not received any special hairdressing such as hair dyeing, perm or styling, etc, within 1 month prior the study; |
| 1. Subject was willing to participate to the study and has provided informed consent forms while fully understanding the study. |
| Exclusion criteria |
| 1. Subject reported being pregnant, nursing or planning recently to do these (only for female); |
| 1. Subject was suffering from severe androgenetic alopecia, alopecia areata, inflammatory cicatricial alopecia or other scalp/ hair diseases; |
| 1. Subject was suffering from mental or psychological diseases; or chronic sleep or emotional control disorder; |
| 1. Subject had used anti-hair loss cosmetics or similar function products or products with the efficacy of promoting hair growth within 3 months prior to the study; |
| 1. Subject had taken or topically applied drugs influencing hair growth within 6 months prior the study; |
| 1. Subject had received hair transplants; |
| 1. Subject had curly hair; |
| 1. Subject was hypersensitive to cosmetics; |
| 1. Subject had participated in other clinical trials within 2 months prior the study; |
| 1. Subject was determined not to be suitable to participate in the study by dermatologist. |

**Table S2.** Demographic data of the model trial.

| **Group** | **Sex (Male/Female)** | **Average age** |
| --- | --- | --- |
| IP | 7/23 | 45.27 ± 8.74 |
| CP | 8/23 | 44.87 ± 11.32 |

Note: IP = Investigation Product, CP = Control Product.

**Table S3.** The results of quantitative hair parameters in different grade (Mean ± SD).

| **Grade** | **Exposed scalp area ratio (%)** | **Dermoscopy parameters** | | | | |
| --- | --- | --- | --- | --- | --- | --- |
|  |  | **Local hair density (****n/cm^2^)** | **Hair diameter (μm)** | **Vellus hairs ratio (%)** | **Terminal hairs**  **ratio (%)** | **Vellus to Terminal hairs ratio (%)** |
| 1 | 43.70 ± 10.29 | 124.43 ± 26.53 | 76.40 ± 7.25 | 16.50 ± 5.45 | 83.50 ± 5.45 | 20.20 ± 7.77 |
| 2 | 33.20 ± 3.48 | 124.12 ± 26.31 | 76.32 ± 10.65 | 15.67 ± 4.77 | 84.33 ± 4.77 | 18.93 ± 6.70 |
| 3 | 28.84 ± 3.94 | 125.07 ± 18.00 | 77.87 ± 7.18 | 13.73 ± 5.75 | 86.27 ± 5.75 | 16.36 ± 7.36 |
| 4 | 20.05 ± 3.53 | 127.40 ± 31.06 | 80.28 ± 4.68 | 13.44 ± 3.35 | 86.56 ± 3.35 | 15.69 ± 4.47 |
| 5 | 15.47 ± 2.33 | 137.26 ± 16.49 | 82.30 ± 6.85 | 13.02 ± 2.71 | 86.98 ± 2.71 | 15.08 ± 3.54 |
| 6 | 13.89 ± 3.88 | 139.96 ± 21.48 | 84.91 ± 3.69 | 12.55 ± 2.19 | 87.45 ± 2.19 | 14.42 ± 2.82 |
| 7 | 11.72 ± 3.07 | 164.59 ± 21.89 | 86.81 ± 4.30 | 11.82 ± 3.23 | 88.18 ± 3.23 | 13.55 ± 4.21 |

Note: The exposed scalp area ratio indicated the proportion of visible scalp area relative to the total analyzed area in the V + F region, the vellus hairs and terminal hairs ratio referred respectively to the percentage of each hair type relative to the total hair number within the analyzed area, and the vellus-to-terminal hair ratio was defined as the numerical ratio of vellus hairs to terminal hairs.

**Table S4-1.** The exposed scalp ratio from different groups

at different time points (Mean ± SD, %).

| **Group**  **Visit** | **IP** | **CP** |
| --- | --- | --- |
| Baseline | 16.55 ± 6.63 | 17.07 ± 5.02 |
| Week 4 | 16.13 ± 6.98 | 16.87 ± 5.79 |
| Week 8 | 15.67 ± 6.73^*^ | 17.37 ± 6.81 |
| Week 12 | 15.51 ± 6.87^**^ | 17.64 ± 5.26 |

Note: IP = Investigation Product, CP = Control Product; significantly different from baseline, **P* < 0.05, ***P* < 0.01.

**Table S4-2.** The net change of exposed scalp ratio from different groups

at different time points (Mean ± SD, %).

| **Group**  **Visit** | **IP** | **CP** |
| --- | --- | --- |
| △Week 4 | -0.42 ± 1.76 | -0.20 ± 2.63 |
| △Week 8 | -0.88 ± 1.94 | 0.29 ± 3.25 |
| △Week 12 | -1.04 ± 1.92^**^ | 0.56 ± 2.28 |

Notes: IP = Investigation Product, CP = Control Product; significantly different from CP, ***P* < 0.01.

**Table S5.** The overall hair density evaluation graded by Sinclair Scale for female and Modified Sinclair Scale for male from different groups at different time points.

| **Group**  **Visit** | **IP**  **(n=24)** | **CP**  **(n-22)** |
| --- | --- | --- |
| Baseline | 1 (1,2) | 1 (1,1) |
| Week 4 | 1 (1,2) | 1 (1,1) |
| Week 8 | 1 (1,2) | 1 (1,1) |
| Week 12 | 1 (1,2) | 1 (1,1) |

Note: IP = Investigation Product, CP = Control Product, n=number of valid subjects; data of 15 subjects were not involved in the statistics analysis because of their alopecia levels less than 1 graded by above scales.

**Table S6.** Overall hair density grading standard in the

“China Safety and Technical Standards for Cosmetics”.

| **Grade** | **Description** |
| --- | --- |
| 1 | Extremely sparse hair density, with clearly visible scalp |
| 2 | Sparse hair density, with easily visible scalp |
| 3 | Slightly sparse hair density, with visible scalp |
| 4 | Medium hair density, with slight visible scalp |
| 5 | Slightly dense hair density, with little visible scalp |
| 6 | Dense hair density, with faintly visible scalp |
| 7 | Extremely dense hair density, with barely visible scalp |


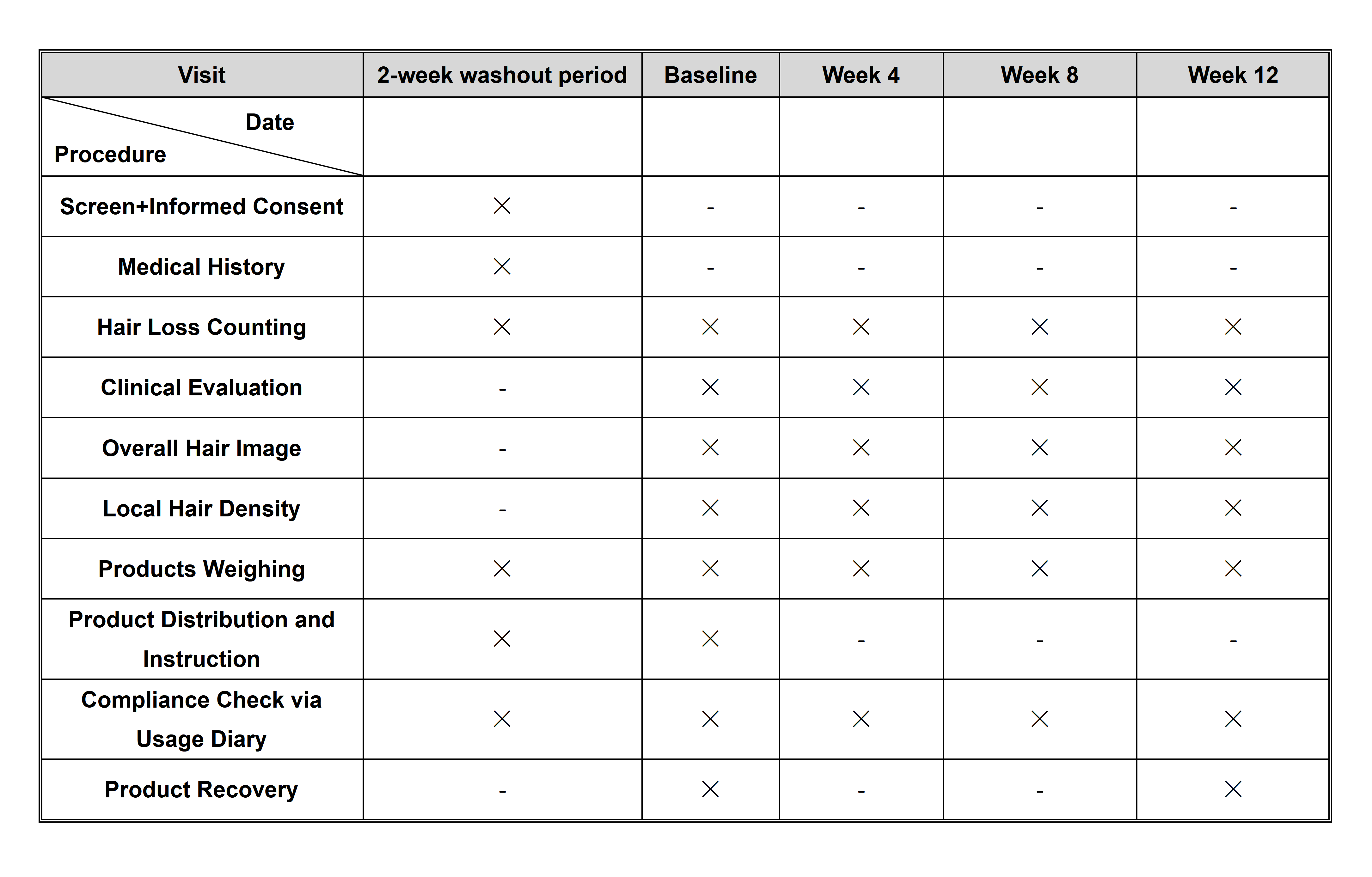


**Figure S1.** Flow chart in the model trial.





**Figure S2.** Instruments results in the model trial. (a) The hair loss counting and (b) The local hair density from different groups at different time points. (c) The net change of hair loss counting and (d) The local hair density from different groups at different time points. Data are presented as Mean ± SD. IP = Investigation Product, CP = Control Product. **P* < 0.05, ***P* < 0.01, ****P* < 0.001.

**
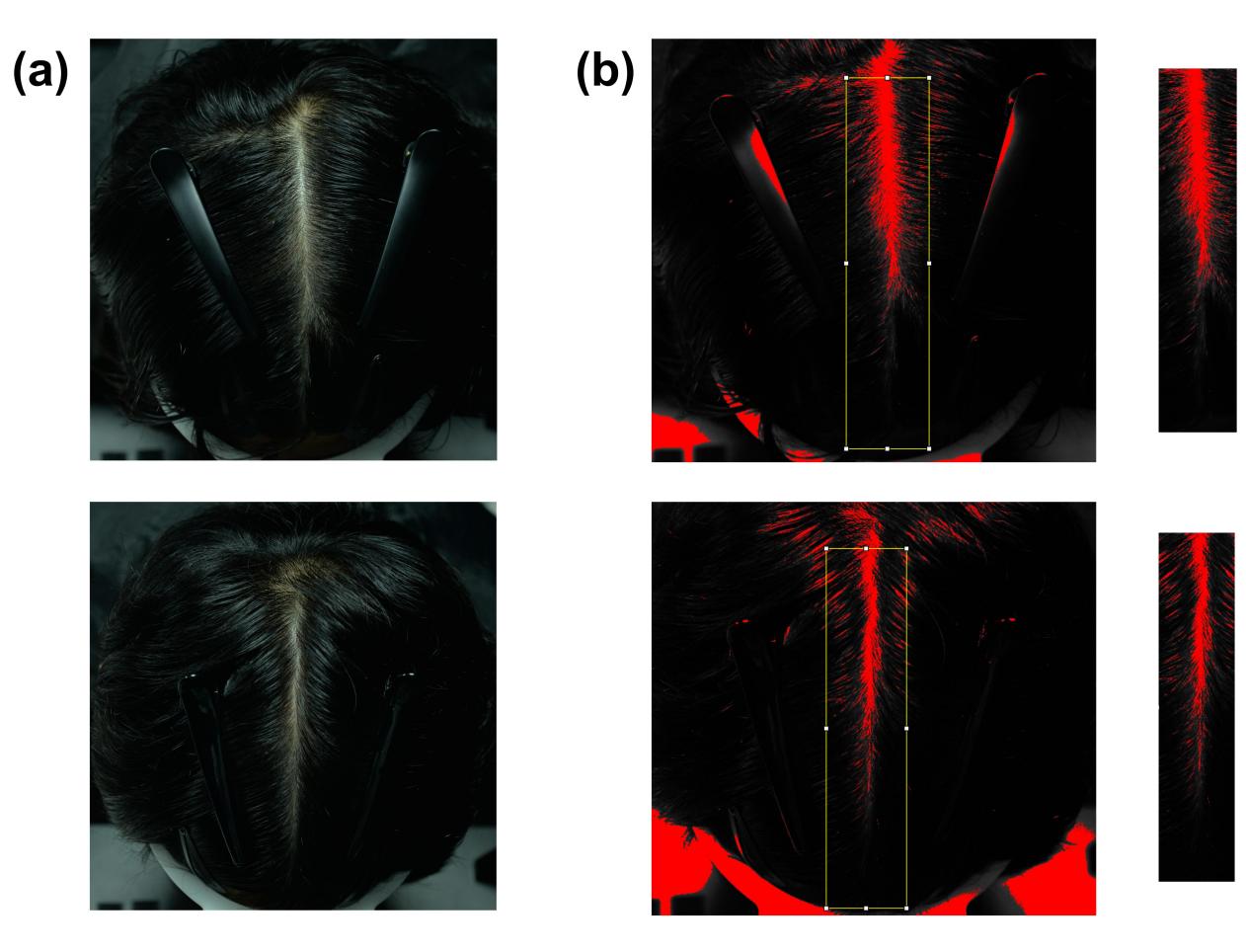
**

**Figure S3.** Representative photographs (subject No. A18) before and after test product application. (a) Digital live photo. (b) Analyzed photo by Image J. (up at Baseline and down at Week 12).


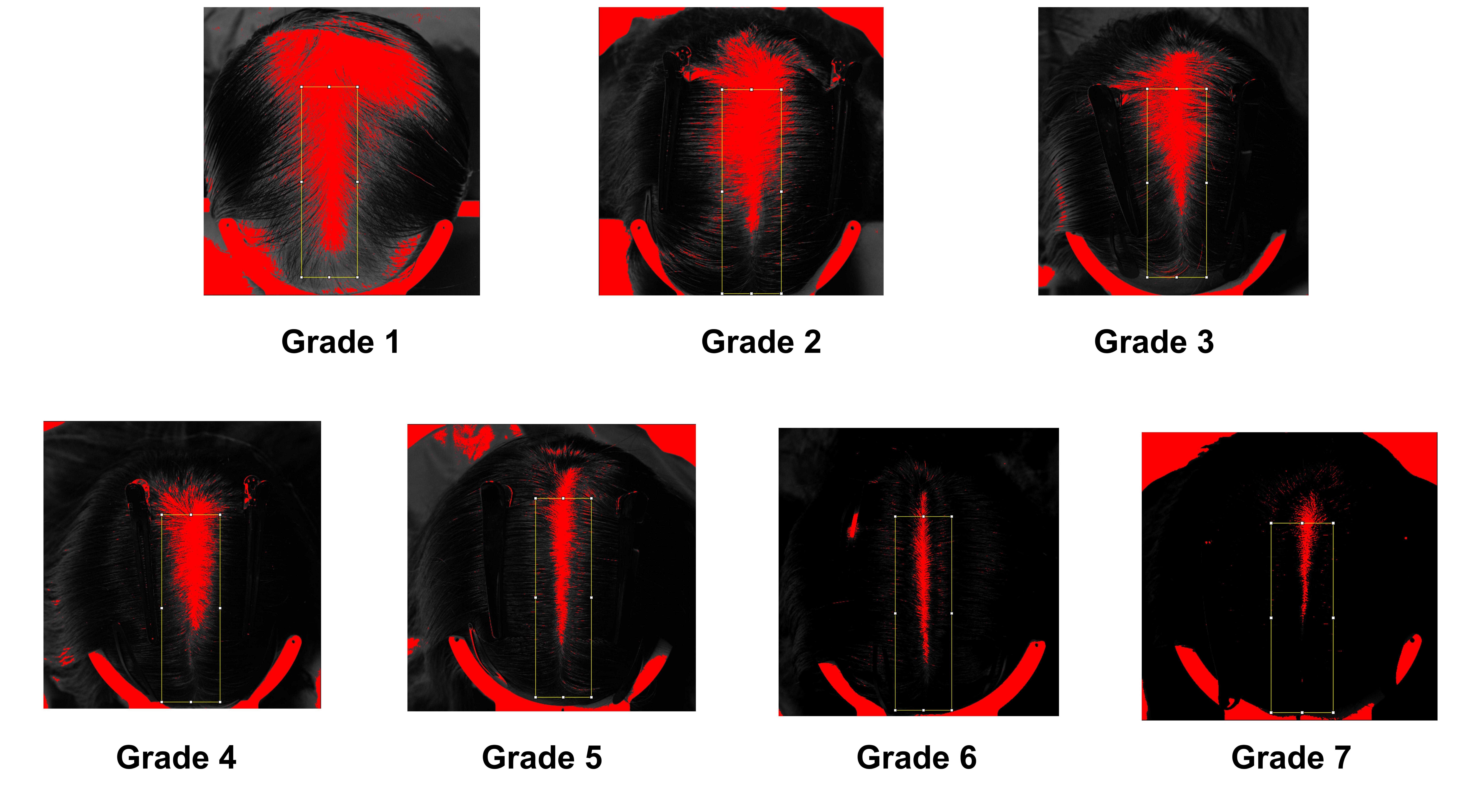


**Figure S4.** The grading scale developed in this study analyzed by Image J.
